# Effects of baclofen and lorazepam on interhemispheric inhibition in humans

**DOI:** 10.1113/EP093963

**Published:** 2026-06-25

**Authors:** Faith C. Adams, Karishma R. Ramdeo, Malaikah Ahmad, Stevie D. Foglia, Chloe C. Drapeau, Mustaali Hussain, Jiyeon Park, Mark A. Tarnopolsky, Aimee J. Nelson

**Affiliations:** ^1^ Department of Kinesiology McMaster University Hamilton Ontario Canada; ^2^ School of Biomedical Engineering McMaster University Hamilton Ontario Canada; ^3^ Department of Pediatrics McMaster Children's Hospital Hamilton Ontario Canada

**Keywords:** baclofen, GABA receptor, interhemispheric inhibition, intervention, lorazepam, motor control, transcallosal connectivity, transcranial magnetic stimulation

## Abstract

Transcallosal projections within the corpus callosum allow for the transfer of information between bilateral primary motor cortices (M1). Interhemispheric inhibition (IHI) is one approach to probe transcallosal communication using transcranial magnetic stimulation (TMS) whereby the motor evoked potential (MEP) elicited by TMS is reduced in amplitude when preceded by a conditioning TMS pulse delivered to the opposite M1. In a double‐blinded, placebo‐controlled study, 24 participants received 2.5 mg of lorazepam (GABA_A_ agonist), 50 mg of baclofen (GABA_B_ agonist) and a placebo in separate sessions. Short‐interval interhemispheric inhibition (SIHI) and long‐interval interhemispheric inhibition (LIHI) recorded from the first dorsal interosseous muscle were quantified before and at peak plasma concentration following drug ingestion. Baclofen significantly reduced SIHI and LIHI while lorazepam had no effect. These findings advance our understanding of the pharmacological basis of transcallosal inhibition in humans.

## Abstract (highlights)

- **What is the central question of this study?**
  What are effects of lorazepam (GABA_A_ receptor agonist), baclofen (GABA_B_ receptor agonist) and a placebo on interhemispheric inhibition in young controls?
- **What is the main finding and its importance?**
  Short‐interval interhemispheric inhibition and long‐interval interhemispheric inhibition are modulated by baclofen but not lorazepam. These results advance our understanding of the mechanisms that govern interhemispheric inhibition between motor cortices.

## 1 INTRODUCTION

Transcallosal projections within the corpus callosum allow for the transfer of information between bilateral primary motor cortices (M1). These projections are essential for producing coordinated motor actions (Morishita et al., 2022; Wahl & Ziemann, 2008), preventing unwanted mirror activity (Beaulé et al., 2012), and fine‐tuning cortical output (Carson, 2020; Merchant & Georgopoulos, 2017). Changes in communication between bilateral M1 may play a role in motor difficulties observed in conditions such as stroke or Parkinson's disease (PD) (Murase et al., 2004; Paparella, De Riggi, Cannavacciuolo, Costa et al., 2023). Characterizing the mechanisms that govern transcallosal communication is therefore essential for understanding motor control and clinically relevant disruptions of interhemispheric communication.

Transcranial magnetic stimulation (TMS) can be used to probe neural circuits underlying transcallosal connectivity. Interhemispheric inhibition (IHI) is one approach whereby the motor evoked potential (MEP) elicited by TMS is reduced in amplitude when preceded by a conditioning TMS pulse delivered to the opposite M1 (Ferbert et al., 1992). The depth of IHI is greatest when two TMS pulses are separated by approximately 10–12 ms and 40–50 ms, referred to as short‐interval interhemispheric inhibition (SIHI) and long‐interval interhemispheric inhibition (LIHI), respectively (Ni et al., 2009). IHI is thought to be mediated via glutamatergic callosal projections that synapse with GABAergic inhibitory interneurons in the contralateral M1 (Daskalakis et al., 2002).

There remain uncertainties surrounding the pharmacological manipulation of SIHI and LIHI. An open label study in healthy controls investigated whether IHI is mediated by GABA_A_ and/or GABA_B_ receptors using the agonists midazolam and baclofen, respectively (Irlbacher et al., 2007). Baclofen increased LIHI with no effect on SIHI, although a placebo control was not included (Irlbacher et al., 2007). Müller‐Dahlhaus et al. (2008) investigated the effects of baclofen and GABA_A_ agonist diazepam on SIHI in a limited sample of control participants (*n* = 8) and observed no effects (Müller‐Dahlhaus et al., 2008). In a placebo‐controlled study, Sommer et al. (2012) examined the effect of the *N*‐methyl‐d‐aspartate (NMDA) receptor antagonist dextrometorphan, the sodium channel blocker carbamazepine, and the GABA_A_ agonist lorazepam, on SIHI and LIHI. Although LIHI was not present at baseline, it emerged in the presence of lorazepam while SIHI decreased only in the presence of carbamazepine (Sommer et al., 2012). In summary, it appears that LIHI may be modulated by drugs that modulate GABA_B_ and GABA_A_ receptors.

The present study aimed to understand whether GABA_A_ and/or GABA_B_ receptor modulation altered SIHI and LIHI. Importantly, this is the first placebo‐controlled study to explore changes in IHI following the delivery of a GABA_B_ agonist. We therefore performed a double‐blinded, placebo‐controlled study where lorazepam, baclofen and placebo were administered in a healthy control population.

## 2 METHODS

### 2.1 Ethical approval

The present study was approved by the Hamilton Integrated Research Ethics Board (no. 16297) and Health Canada. The research conformed to the standards set by the *Declaration of Helsinki* except for registration in a database. All participants provided their written informed consent prior to participation in the study.

### 2.2 Participants

Twenty‐four healthy, right‐handed individuals (22 ± 2.5 years, 7 females, 17 males) participated in three sessions at least 7 days apart (15 ± 14 days). All participants were screened for contraindications to TMS, baclofen and lorazepam using screening questionnaires. Right‐handedness was confirmed by the modified handedness questionnaire (Oldfield, 1971). Participants were excluded if they were taking any prescribed medication, except for hormonal contraceptives that were permitted. Participants taking recreational drugs were excluded.

### 2.3 Electromyography

Surface electromyography (EMG) electrodes (9 mm Ag–AgCl) were placed over the first dorsal interosseous (FDI) muscle of both hands. A ground electrode was placed at the styloid process of the right wrist. All EMG recordings were amplified 1000× (model 2024F; Intronix Technologies Corporation, Bolton, ON, Canada), band‐pass filtered between 20 Hz and 2.5 kHz and digitized at 5 kHz using an analog‐to‐digital converter (Power 1401; Cambridge Electronic Design, Cambridge, UK). Data were analysed using commercial software (Signal, version 7.02; Cambridge Electronic Design).

### 2.4 Transcranial magnetic stimulation

TMS was performed with a 50 mm figure‐of‐eight branding coil connected to a Magstim 200^2^ stimulator (Magstim, Whitland, UK). The coil was positioned over M1 at the location that elicited a MEP in the contralateral FDI muscle (i.e. the motor hotspot). The coil was oriented at a 45‐degree angle from the sagittal plane to induce a posterior–anterior current in the cortex. The location and orientation of each coil were registered each session using Brainsight Neuronavigation (Rogue Research, Montreal, QC, Canada) (Foglia et al., 2024) at the start of each session. To determine baseline cortical excitability, resting motor threshold (RMT) (Siebner & Rothwell, 2003) was determined using TMS_MTAT_2.0 freeware (http://clinicalresearcher.org/software.htm). This program employs a maximum likelihood parameter estimation by sequential testing and uses a probabilistic method of estimating RMT (Sen et al., 2017). The stimulus intensity was set to 37% of the maximum stimulator output (MSO) and 20 TMS pulses were delivered over the FDI hotspot to determine the RMT. RMT was performed for each hemisphere.

Assessment of IHI was performed using paired‐pulse TMS using two separate coils. A conditioning stimulus (CS) was delivered over M1 in the right hemisphere, followed by a test stimulus (TS) over M1 in the left hemisphere, both at the motor hotspot of the FDI muscle. IHI was assessed at two different latencies. SIHI was assessed by using a 10 ms interstimulus interval (ISI) between CS and TS pulses (CSTS_10_) (Ni et al., 2009; Turco et al., 2019). LIHI was assessed using an ISI of 50 ms (CSTS_50_) (Ni et al., 2009). The CS was delivered at 130% RMT before drug ingestion, and this same intensity was used after drug ingestion (i.e., unadjusted CS). The TS was delivered at an intensity sufficient to elicit a peak‐to‐peak MEP amplitude of 1 mV in the contralateral muscle at rest before and adjusted to elicit a 1 mV MEP after the intervention. IHI was examined in the FDI muscle of the right hand. Twenty CSTS_10_, 20 CSTS_50_ and 20 TS‐alone trials were pseudo‐randomly delivered in the block of trials. The magnitude of IHI was quantified as the ratio of the mean MEP amplitude obtained during CSTS_10_ and CSTS_50_ to the TS alone (IHI = CSTS_10_/TS; CSTS_50_/TS).

### 2.5 Experimental design

**FIGURE 1:**
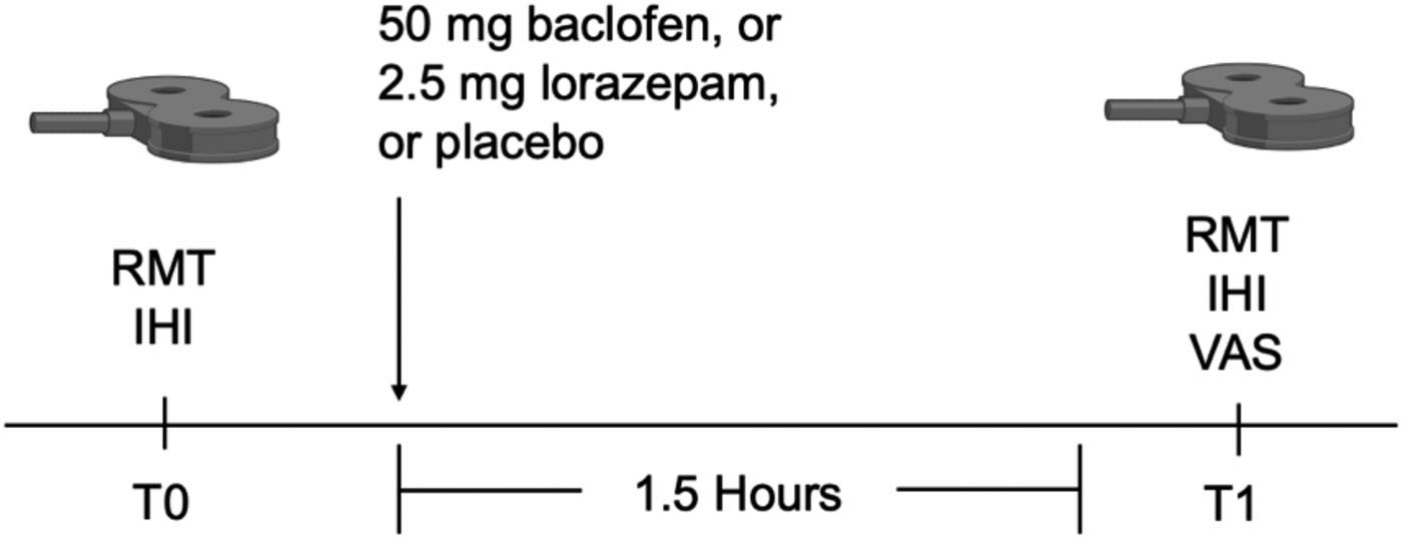
Experimental timeline. Baseline (T0) measures of IHI and RMT were acquired prior to drug ingestion. Individuals were randomized to ingest either 50 mg of baclofen, or 2.5 mg of lorazepam, or placebo. Following 1.5 h (T1), RMT, IHI and VAS were acquired. IHI, interhemispheric inhibition; RMT: resting motor threshold; T0, baseline, pre‐drug administration; T1, post‐drug administration; VAS, visual analogue scale.

Participants attended three sessions where they were administered either baclofen (50 mg) (Irlbacher et al., 2007), or lorazepam (2.5 mg) (Turco et al., 2018) or a placebo. The randomization schedule included six intervention permutations and was prepared by McMaster University Medical Centre Pharmacy who also prepared all drug kits. All drugs were formulated to be identical in appearance to ensure blinding across participants and experimenters. RMT and IHI were acquired prior to drug ingestion at baseline (T0), and at 1.5 h after drug ingestion (T1) based on the peak plasma concentration of the drugs (Turco et al., 2018; Ziemann et al., 1996). During the 1.5 h period between T0 and T1, participants remained in the lab and were not permitted to eat or sleep. To evaluate the sedative effects of lorazepam and baclofen, a visual analogue scale (VAS) was performed by the participant and two independent experimenters at T1. This scale consisted of a 10 cm line with 0 cm indicating that the participant was ‘alert’ and 10 cm indicated ‘extremely sedated’ (Turco et al., 2018). All sessions started in the morning and all experiments and analyses were performed by experimenters who were blinded to the treatment allocation. The experimental timeline is illustrated in Figure 1.

### 2.6 Statistical analysis

To avoid contamination of the MEP by background muscle activity, EMG trials were discarded if the peak‐to‐peak amplitude of the signal 100 ms before the TMS artifact was greater than 50 µV. Normality for all variables were assessed using the Shapiro–Wilks test and outliers were assessed using Grubbs’ test. A one‐way ANOVA with factor Drug (lorazepam, baclofen, placebo) was performed to determine whether baseline RMT was different across drug sessions. To establish the presence of significant IHI at baseline, a two‐way ANOVA with within‐subjects factors Pattern (CS_10_, CS_50_, TS) and Drug (lorazepam, baclofen, placebo) was performed on T0 data.

To investigate the effect of drug, a two‐way ANOVA was performed on RMT data using the within‐subjects factors Time (T0, T1) and Drug (lorazepam, baclofen, placebo). A three‐way ANOVA was performed on IHI data using within‐subject factors ISI (10 ms, 50 ms), Time (T0, T1), and Drug (lorazepam, baclofen, placebo). For all analyses, *P* < 0.05 was considered statistically significant. In the event of a significant finding, *post hoc* testing was performed using Tukey's honest significant difference test.

VAS scores were assessed using the Mann–Whitney *U*‐test between the two experimental raters. VAS scores were not different between raters, and therefore the scores were subsequently averaged for each visit. Two separate Freidman's tests were performed to determine if the participants and the averaged experimental raters VAS scores differed across drugs (3 levels: lorazepam, baclofen, placebo). Additionally, Mann–Whitney *U*‐tests were performed to compare whether the participants and averaged experimental raters VAS score were different following ingestion of each drug. To determine whether changes in IHI were related to sedation levels after drug ingestion, three separate Spearman's rho correlations were performed for each drug condition using the percentage change in IHI_AVG_ compared to the average VAS scores from the participants and experimenters. For all analyses, *P* < 0.05 was considered statistically significant.

Reliability statistics were performed on the placebo data only to determine the measurement error associated with repeated testing of IHI. Absolute reliability was determined for the placebo intervention for each dependent measure using the standard error of measurement (SEM_eas_) values (SEM_eas_ = √mean squared error). The SEM_eas_ was used to determine the smallest detectable change (SDC) at an individual level (SDC_ind_ = SEM_eas_ × 1.96 × √2), which was used to calculate SDC_group_ (SDC_group_ = SDC_ind_/√*n*).

## 3 RESULTS

No adverse events were observed following administration of any drug and there was no attrition. Data from one participant was removed due to technical issues yielding a total sample size of 23. The data passed normality testing and outliers were not detected. Table 1 presents group‐averaged means with standard deviation. RMT was not different across drug sessions at baseline (*F*_(2,68)_ = 0.004, *P* = 0.996). To examine whether inhibition was present at baseline, two‐way ANOVA revealed a main effect of Pattern (*F*_(2,44)_ = 47.49, *P* < 0.001) such that CSTS_10_ and CSTS_50_ were significantly suppressed relative to TS (*P* < 0.001), and CSTS_10_ and CSTS_50_ were not different from each other (*P* = 0.242). At baseline, there was no main effect of Drug (*F*_(2,44)_ = 1.64, *P* = 0.205) or interactions (*F*_(4,88)_ = 0.26, *P* = 0.901), suggesting that baseline inhibition did not differ between days.

**TABLE 1:** Group‐averaged data (means ± SD) measured before (T0) and after (T1) administration of lorazepam, baclofen and placebo.

	Lorazepam		Baclofen		Placebo	
	T0	T1	T0	T1	T0	T1
RMT (%MSO)	37.30 ± 6.08	37.83 ± 6.43	37.39 ± 6.47	38.00 ± 6.43	37.48 ± 6.30	37.61 ± 6.72
1 mV (%MSO)	44.78 ± 7.85	45.61 ± 8.32	44.65 ± 6.69	45.22 ± 7.18	44.74 ± 8.29	44.83 ± 8.90
TS (mV)	0.96 ± 0.23	0.92 ± 0.22	0.91 ± 0.19	1.02 ± 0.29	0.99 ± 0.24	1.00 ± 0.23
CS_10_ (mV)	1.61 ± 1.49	1.50 ± 1.50	1.40 ± 1.52	1.37 ± 1.60	1.85 ± 2.25	1.92 ± 1.89
CS_50_ (mV)	1.53 ± 1.39	1.49 ± 1.48	1.47 ± 1.61	1.38 ± 1.64	1.86 ± 2.16	2.00 ± 1.86
CSTS_10_/TS	0.66 ± 0.21	0.69 ± 0.24	0.62 ± 0.24	0.75 ± 0.30	0.65 ± 0.27	0.61 ± 0.28
CSTS_50_/TS	0.72 ± 0.20	0.68 ± 0.28	0.67 ± 0.25	0.84 ± 0.25	0.72 ± 0.24	0.64 ± 0.22

*Note*: CS amplitudes (mV) were measured in the left FDI muscle. TS amplitudes (mV) were measured in the right FDI muscle. Abbreviations: %MSO, maximal stimulator output; CS_10_, conditioning stimulus delivered 10 ms prior to the TS; CS_50_, conditioning stimulus delivered 50 ms prior to the TS; CSTS_10_/TS, short‐interhemispheric inhibition; CSTS_50_/TS, long‐interhemispheric inhibition; RMT, resting motor threshold; TS, testing stimulus.

### 3.1 Effects of drug intervention

RMT data revealed no effect of Time (*F*_(1,22)_ = 1.19, *P* = 0.288), Drug (*F*_(2,44)_ = 0.06, *P* = 0.940) or their interaction (*F*_(2,44)_ = 0.27, *P* = 0.763). This is consistent with previous findings that report no effects of lorazepam (Boroojerdi et al., 2001; Di Lazzaro et al., 2000; Turco et al., 2018; Ziemann et al., 1996) and baclofen (Inghilleri et al., 1996; McDonnell et al., 2007; Turco et al., 2018) on measures of RMT.

**FIGURE 2:**
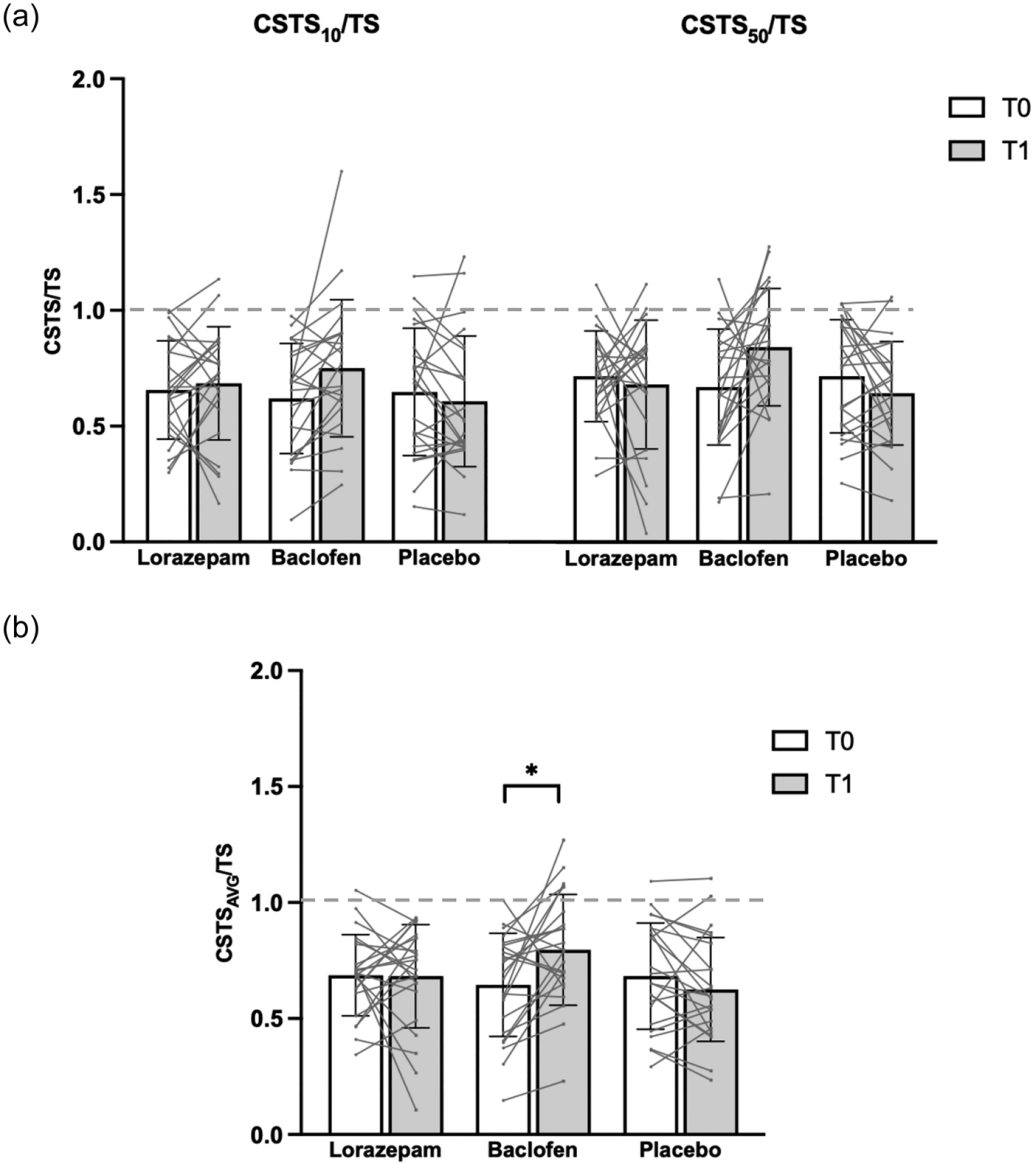
Interhemispheric inhibition following the administration of lorazepam, baclofen and placebo. (a) IHI (mean ± SD) expressed as a ratio of the conditioned MEP (CSTS_10_ and CSTS_50_) to the unconditioned MEP (TS) before (T0) and after (T1) drug administration. (b) IHI (mean ± SD) expressed as a ratio of the conditioned MEP (CSTS_Averaged_) to the unconditioned MEP (TS) before (T0) and after (T1) drug administration. Data points below 1 represent inhibition whereas points above 1 represent facilitation. *P < 0.05, Three‐way ANOVA. *n* = 23.

Figure 2a plots the ratio of CSTS/TS for each of CSTS_10_ and CSTS_50_ for each drug intervention. ANOVA revealed no effect of ISI (Table 2) and a significant interaction of Time × Drug (*F*_(2,44)_ = 5.39, *P* = 0.008) plotted in Figure 2b. *Post hoc* analyses revealed a significant decrease in IHI following baclofen only (T0: 0.65 ± 0.22, T1: 0.8 ± 0.24, *P* = 0.002). The average decrease following baclofen ingestion was 23.1%. Of the 23 participants, 74% demonstrated a reduction in IHI following baclofen. ANOVA did not reveal any other statistical main effects or interactions.

**TABLE 2:** Three‐way ANOVA statistics.

Effect	Statistic	*P*	Effect size (η_p_^2^)
Time	*F*_(1,22)_ = 1.25	0.274	0.05
ISI	*F*_(1,22)_ = 1.88	0.184	0.08
Drug	*F*_(2,44)_ = 1.13	0.331	0.05
**Time × drug**	***F*_(2,44)_ = 5.39**	**0.008**	**0.20**
ISI × time	*F*_(1,22)_ = 0.50	0.485	0.02
ISI × drug	*F*_(2,44)_ = 0.64	0.528	0.03
Time × ISI × drug	*F*_(2,44)_ = 0.92	0.403	0.04

Bolded values indicate statistically significant effects (*P* < 0.05).

The mean VAS score reported by participants was significantly greater following lorazepam (5.0 ± 2.1) compared to baclofen (2.6 ± 2.3) and placebo (2.0 ± 1.8) (Friedman's test, *P* < 0.001). VAS scores were not significantly different between experimenters (Mann–Whitney *U*‐test, placebo: *P* = 0.796; baclofen: *P* = 0.767; lorazepam: *P* = 0.941) and were subsequently averaged. Similar to the participants, experimenter averaged VAS scores were significantly greater following lorazepam (4.4 ± 2.1) compared to baclofen (2 ± 1.9) and placebo (1.3 ± 1.3) (Friedman's test, *P* < 0.001). The average VAS score from the experimenters was not different from the participants’ reported VAS score (Mann‐Whitney U test, placebo: *P =* 0.215; baclofen: *P* = 0.267; lorazepam: *P *= 0.408), and therefore scores from experimenters and participants were averaged for subsequent correlational analyses. No correlations between the average VAS scores and percentage change of IHI (averaged across CSTS_10_, CSTS_50_) were observed for any drug (placebo: *P* = 0.921, *r* = 0.02; baclofen: *P* = 0.091, *r* = 0.36; lorazepam: *P* = 0.595, *r* = −0.12) indicating that sedation was not associated with changes in IHI.

### 3.2 Reliability statistics

Absolute reliability was determined using the data acquired from the placebo condition to assess the measurement error that occurs due to repeated testing of an individual (Table 3). Group‐level reliability expressed as %SEM_eas_ was >10% for CSTS_10_/TS, CSTS_50_/TS and CSTS_AVG_/TS demonstrating a large amount of measurement error (Schambra et al., 2015). Based on the SDC_group_ of the CSTS_AVG_/TS, we calculated the real physiological reduction in IHI to be 12.3% (23.1% − 10.8%) following baclofen ingestion.

**TABLE 3:** Reliability statistics.

	SEM_eas_	%SEM_eas_	SDC_group_
CSTS_10_/TS (%)	14.4	23.5	13.4
CSTS_50_/TS (%)	14.9	21.8	12.6
CSTS_AVG_/TS (%)	12.3	18.8	10.8

Abbreviations: %SEM_eas_, percentage standard error of measurement; SDC_group_, smallest detectable change at the group level; SEM_eas_, standard error of measurement.

## 4 DISCUSSION

The present study examined the pharmacological influence of GABA_A_ and GABA_B_ receptor agonists on the depth of IHI. Our analysis did not reveal any distinctions between CSTS_10_ and CSTS_50_. Therefore, our novel finding demonstrates a reduction in IHI following baclofen, which encompasses both SIHI and LIHI. Considering the SDC_group_, the reduction in IHI is ∼12.3%. We discuss these findings and possible neural mechanisms below.

### 4.1 Effects of baclofen on IHI

Two studies examined the effects of 50 mg of baclofen on SIHI, and both reported no significant changes (Irlbacher et al., 2007; Müller‐Dahlhaus et al., 2008). Irlbacher et al. (2007) observed a non‐significant trend towards increased SIHI following baclofen administration. Their methodology closely resembled the present study as SIHI was probed at an ISI of 10 ms and demonstrated comparable baseline depth of inhibition (∼ 40%), although a placebo control was not included. Müller‐Dahlhaus et al. (2008) reported no change in SIHI, which may be due to the limited sample size (*n* = 8). Collectively, these methodological differences may account for the discrepancies between previous and present results.

Irlbacher et al. (2007) observed a ∼20% increase in LIHI following baclofen. An important distinction is the difference in the delivery of the CS. In the present study, the CS intensity (130% RMT) was established at baseline (T0), and this intensity was maintained when IHI was measured following drug ingestion (T1). Therefore, the CS is considered unadjusted in this approach, which permits a direct assessment of drug‐induced changes in transcallosal activity. This approach is consistent whereby the CS intensity is unadjusted following various interventions for measures of short‐interval intracortical inhibition (SICI) (Daskalakis et al., 2006; Di Lazzaro et al., 2005, 2006; Huang et al., 2005) and IHI (Hinder et al., 2010; Perez & Cohen, 2008). In contrast, Irlbacher et al. (2007) adjusted both CS and TS intensities following drug administration. Such adjustments may alter neuronal recruitment at T1 and may have changed the opportunity to observe drug‐induced changes in the transcallosal connections. Future work should examine IHI after pharmacological administration whereby the CS and TS were both unadjusted.

**FIGURE 3:**
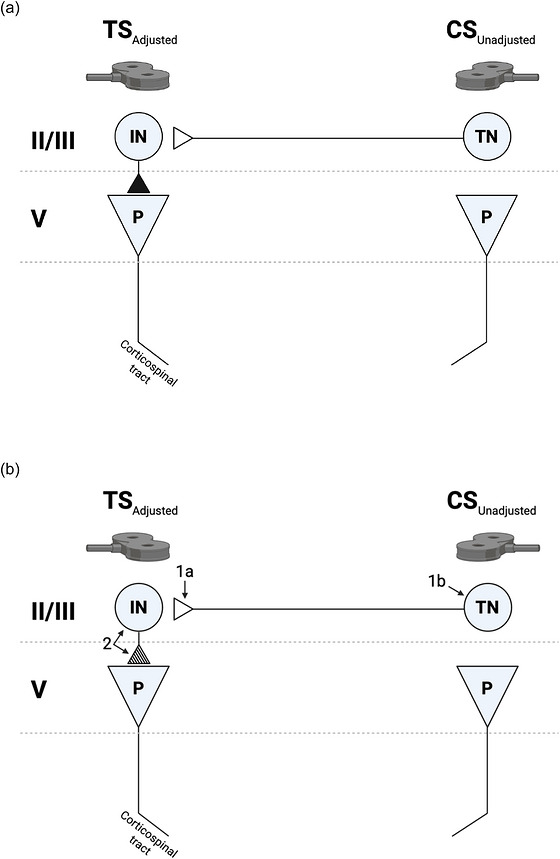
Proposed model of the effects of baclofen on IHI. Simplified model of the neural connections that may mediate IHI. (a) IHI circuit. The CS is delivered to right hemisphere M1, and the TS is delivered to the left hemisphere M1. The CS pulse activates TN, which synapse with IN in left M1, which, in turn, synapse with P neurons contributing to the corticospinal tract. In the absence of pharmacological agents, the CS inhibits the output of the TS. (b) Mechanisms of baclofen on IHI. Baclofen acting at sites 1a and 1b would lead to reduced excitatory activity of the transcallosal projection, which reduces the inhibitory activity of the GABAergic interneuron, resulting in less inhibition exerted on pyramidal cells. Baclofen binding to site 2 results in less inhibition exerted on pyramidal cells, leading to a net reduction of IHI. In the presence of baclofen, the CS inhibition of TS is reduced. Hatched triangle indicates less inhibition. CS: conditioning stimulus; IN: inhibitory interneurons; P: pyramidal neurons; TN: transcallosal neurons; TS: test stimulus.

IHI is thought to depend on glutamatergic transcallosal projections that synapse onto inhibitory interneurons in the contralateral M1 (Daskalakis et al., 2002), resulting in a reduction of corticospinal output (Figure 3a). GABA_B_ receptors are expressed both pre‐ and postsynaptically, allowing baclofen to act at both sites. By adjusting the TS at T1 to account for drug‐induced changes in corticospinal excitability, this allows us to infer that changes in IHI depth result from the pre‐ and postsynaptic modulation of transcallosal projections. In mouse models, baclofen acting at presynaptic GABA_B_ receptors, reduces presynaptic calcium influx (Chalifoux & Carter, 2010), which results in a decrease of neurotransmitter release. In the context of IHI, activation of GABA_B_ receptors on presynaptic glutamatergic callosal neurons is expected to attenuate excitation of inhibitory interneurons (in the hemisphere receiving the TS), which reduces the inhibition of pyramidal cells, evoking an increase of the CSTS amplitude and resulting in a reduction of IHI (Figure 3b, 1). Additionally, baclofen may act directly on the GABAergic interneurons in the hemisphere receiving the TS, similarly leading to disinhibition of the pyramidal neurons, and reducing IHI (Figure 3b, 2). It is important to note, we adjusted the TS intensity at T1, thereby controlling any changes that might have occurred within the contralateral hemisphere, making this mechanism unlikely to explain the present findings. Consistent with this, neither the RMT nor the stimulus intensity required to evoke a 1 mV MEP differed between T0 and T1, suggesting that baclofen did not alter corticospinal excitability. In contrast, previous reports show a decrease in corticospinal excitability (CSE) following baclofen (Johnstone et al., 2021); however, the study used a lower dosage of baclofen, which may induce differential corticospinal effects, and it is unknown how the dosage of baclofen influences corticospinal excitability. Collectively, the observed reduction in IHI appears to be the result of baclofen acting to modulate transcallosal projections.

### 4.2 Effects of lorazepam on IHI

In the present study, 2.5 mg of lorazepam did not significantly modulate SIHI or LIHI. This aligns with previous findings that neither lorazepam (Ziemann et al., 1996) nor diazepam (Müller‐Dahlhaus et al., 2008), nor midazolam (Irlbacher et al., 2007) modulate IHI. In contrast, Sommer et al. (2012) administered 2 mg of lorazepam and observed a significant increase in LIHI. One difference that may account for this discrepancy is that inhibition was not observed at baseline (Sommer et al., 2012) suggesting the administration of a GABA_A_ agonist may facilitate the emergence of inhibition. In contrast, when inhibition is already present pre‐drug, as observed here, additional GABA_A_ receptor potentiation does not appear to produce changes in inhibition. This pattern may reflect interindividual variability in baseline IHI, which may influence the extent to which lorazepam modulates IHI.

Although we did not observe an effect following administration of lorazepam, other drugs targeting alternative subunits may observe modulation of IHI. Lorazepam binds to the A chain of the β2 subunit and E chain of the γ2 subunit of the GABA_A_ receptor (Roy et al., 2024) and has been shown to increase SICI (Di Lazzaro et al., 2005). It is possible, that different GABA_A_ agonists, such as bromazepam or clonazepam, which have affinity for the C chain of the β2 subunit and D chain of α2 subunit (Roy et al., 2024) may modulate IHI.

### 4.3 Functional relevance of SIHI and LIHI

Several studies, including the present work, suggest that both SIHI and LIHI may not be as distinct as previously thought. We demonstrate that both SIHI and LIHI are reduced by baclofen and not lorazepam. Further, SIHI and LIHI are strengthened by increasing CS intensities, and the conditioning coil orientation has no effect on either measure (Ni et al., 2009). SIHI and LIHI are both increased after bilateral transcranial alternating current stimulation (tACS) (Lebihan et al., 2025) and reduced after 1 Hz repetitive TMS (Pal et al., 2005). Further, both circuits have been shown to be reduced in the presence of a unilateral contraction contralateral to the TS (Nelson et al., 2009; Turco et al., 2019) and negatively correlated with cortical territory as measured by motor maps (Turco et al., 2019).

Other research suggests that SIHI and LIHI are indeed distinct based on differential modulation of each circuit. For example, LIHI is elicited by lower intensity stimuli than SIHI (Ni et al., 2009). Task‐dependent modulation further dissociates these measures. For example, during unilateral contraction of the hand ipsilateral to the TS, SIHI is significantly increased, whereas LIHI remains unchanged (Uehara et al., 2014). Similarly, during a fine motor task, SIHI increases, and LIHI decreases when assessed from the active to resting M1, suggesting functional differences between SIHI and LIHI (Morishita et al., 2012). In addition, following continuous theta burst stimulation over primary somatosensory cortex, the magnitude of SIHI increases, whereas LIHI remains unchanged (Zapallow et al., 2013).

IHI is a neurophysiological tool to probe how inhibitory processes shape and refine motor output across hemispheres. IHI can be characterized as an index of surround/lateral inhibition whereby inhibitory networks sculpt cortical activity to enhance the precision of voluntary movement (Carson, 2020). Additionally, IHI is dynamically modulated according to behavioral context. For example, during unilateral movement, IHI is increased from the active to the rest hemisphere (Vercauteren et al., 2008). Additionally, in bimanual tasks, greater IHI may be associated with reduced capacity for interhemispheric cooperation resulting in poor performance on bimanual tasks and/or tasks that require high levels of coordination (Fling & Seidler, 2012). In motor learning, the successful transfer of a visuomotor task between the upper limbs is associated with the reduction of IHI. That is, the better the transfer of the motor task from the dominant to the non‐dominant hand, the greater the reduction of SIHI recorded from the non‐dominant hand (Paparella, De Riggi, Cannavacciuolo, Colella et al., 2023). Together, these findings indicate that IHI may be associated with unilateral movement, bilateral coordination and motor learning. Clinically, reduced IHI has been reported in PD (Spagnolo et al., 2013; Paparella, De Riggi, Cannavacciuolo, Costa et al., 2023) multiple sclerosis (Hagen et al., 2024), amyotrophic lateral sclerosis (Karandreas et al., 2007), dystonia (Beck et al., 2009; Nelson et al., 2010) and stroke (Mirdamadi et al., 2023).  In individuals living with PD, the magnitude of transcallosal inhibition is imbalanced between hemispheres whereby the hemisphere contralateral to the most affected limb exerts increased inhibitory control of the opposite hemisphere. This physiological imbalance in transcallosal inhibition is associated with the severity of bradykinesia, as measured using a sequential tapping task (Paparella, De Riggi, Cannavacciuolo, Costa et al., 2023). Further, individuals with writer's cramp show a significant reduction in IHI recorded from the affected hand, relative to IHI recorded from the unaffected hand. This asymmetry suggests that IHI may have a valuable role for assessing motor impairments in this condition (Nelson et al., 2010). In stroke, both increased IHI (Boddington & Reynolds, 2017) and reduced IHI (Mirdamadi et al., 2023) are reported for the hand contralateral to the lesioned M1. Importantly, greater differences in IHI between rest and active contraction conditions are associated with more severe motor impairment indicating that modulation of IHI is related to motor function of the upper limb in stroke (Mirdamadi et al., 2023). The reduction of IHI following baclofen administration observed herein reflects that GABAergic mechanisms may contribute to disruptions in this pathway and may be associated with motor impairment. Individuals taking baclofen for symptom management may exhibit altered IHI compared to those not taking the medication. Such modulation of IHI may be related to motor behaviour, particularly in clinical populations where IHI is already modulated. As a result, the use of baclofen may represent an important confounding factor when interpreting IHI. Future work should examine the relationship between baclofen‐induced changes in IHI and their functional behavioural consequences.

Collectively, these findings support the view that although SIHI and LIHI share overlapping pharmacological GABA_B_‐mediated mechanisms, the differences in task‐dependent modulation suggest distinct functional roles between the circuits. Further research is needed to expose the behavioural correlates of SIHI and LIHI to improve our understanding of these circuits.

### 4.4 Limitations and future directions

Both participants and experimenter raters reported higher VAS scores following lorazepam administration compared to baclofen or placebo indicating perceived drug effects. Although participants were unaware of the specific drug they received, due to this study including two pharmacological agents, we cannot exclude the possibility of partial unblinding. Additionally, this sample was predominantly male participants. Previous work has demonstrated that females have increased IHI compared to males (Gennaro et al., 2004), which is thought to result from greater volume of anterior callosal connectivity in females (Davatzikos & Resnick, 1998). Future pharmacology work examining interhemispheric inhibition should include an equal number of men and women to determine if pharmacological modulation differs between sex. Additionally, future work should examine other pharmacological agents that may have affinity for different subunits of the GABA_A_ receptors to determine if IHI is GABA_A_ modulated.

### 4.5 Conclusion

We have demonstrated that SIHI is modulated by GABA_B_ receptor activity. SIHI and LIHI were reduced by baclofen. Furthermore, SIHI and LIHI are not influenced by lorazepam. These findings advance our understanding of the pharmacological basis of transcallosal inhibition in humans.

## AUTHOR CONTRIBUTIONS

Faith C. Adams and Aimee J. Nelson conceptualized and designed the study. Faith C. Adams, Karishma R. Ramdeo, Chloe C. Drapeau, Malaikah Ahmad, Jiyeon Park, and Mustaali Hussain were involved in the investigation and acquisition of data. Faith C. Adams, Karishma R. Ramdeo, Stevie D. Foglia, and Aimee J. Nelson were involved in the formal analysis and interpretation of the work. Faith C. Adams, Malaikah Ahmad, and Chloe C. Drapeau were responsible for visualization. Faith C. Adams, Karishma R. Ramdeo, Chloe C. Drapeau, Malaikah Ahmad, Mustaali Hussain, Stevie D. Foglia, Jiyeon Park, Mark A. Tarnopolsky, and Aimee J. Nelson were responsible for original draft and editing.

## CONFLICT OF INTEREST

None declared.

## GENERATIVE AI STATEMENT

No generative AI tools were used in the preparation of this manuscript.

## Data Availability

All data will be made accessible via a data repository upon request.
